# Evaluating school health practices in Pennsylvania: Development, results, and insights of the revised 2024–2025 healthy champions assessment

**DOI:** 10.1016/j.pmedr.2026.103386

**Published:** 2026-01-14

**Authors:** Allison M. Linton, Alicia M. Hoke, Lindsay A. Aluquin, Erik B. Lehman, Deepa L. Sekhar

**Affiliations:** a Penn State College of Medicine, Pediatrics, 700 HMC Crescent Road, Hershey, PA 17033, United States of America; b Penn State College of Medicine, Public Health Sciences, 700 HMC Crescent Road, Hershey, PA 17033, United States of America

**Keywords:** School health, Assessment, Prevention, Wellness, Public health, Evaluation

## Abstract

**Objective:**

This manuscript describes updates to the Healthy Champions school health assessment, revised to align with evolving Pennsylvania and national policies. For the 2024–2025 program year, the tool was updated to reflect best practices that emerged since COVID-19, and current trends in school health.

**Methods:**

Data were collected via the web-based Healthy Champions assessment, open from April–June 2024. The assessment, including five sections – Physical Activity and Education; Nutritional Environment; Counseling and Psychological Services; Health Services and Education; and School Health Environment and Culture, surveyed schools on best practices and regulatory standards. Responses were scored as section scores and analyses examined differences by respondent role type.

**Results:**

Among the 328 responding schools, two-thirds (*n* = 216, 65.9%) meet or exceed standards in all five sections. The highest scores were observed in Health Services and Education, and Psychological Services and Education, while lowest scores fell in the School Health Environment and Culture section.

**Conclusions:**

Despite high reports of adherence to standards, findings reveal challenges in achieving comprehensive practices. Strengthening school health councils and wellness policies, in concert with improved policies, represent key opportunities to enhance school health in Pennsylvania.

## Highlights

• Pennsylvania schools report meeting or exceeding standards across health domains.
• Schools consistently report achieving standards in basic health practices.
• Reported practices reveal gaps in achieving comprehensive school wellness.
• School health best practices and standards have evolved since the COVID-19 pandemic.

## 1 Introduction

To address the complex health challenges affecting youth (CDC – YRBS Explorer, 2023; Samji et al., 2022; Sanyaolu et al., 2019), schools must cultivate environments that support holistic student health and academic success (CDC - Healthy Schools, 2024). The Centers for Disease Control (CDC), Whole School, Whole Community, Whole Child Model (WSCC) offers a framework for school health efforts (CDC - Whole School, 2026). The WSCC model aligns health and education goals through a community-oriented approach that centers on students' cognitive, physical, and socioemotional development (Lewallen et al., 2015).

The 2004 reauthorization of the Child Nutrition and WIC Act required wellness policies for schools participating in the National School Lunch/Breakfast Programs (Local School Wellness Policy, 2025). Subsequently, school health assessments became necessary tools for meeting standards and to identifying strengths and gaps in a school's health environment. Tools like the Wellness School Assessment Tool (WellSAT) or CDC's School Health Index (SHI) are just two examples of established tools for measuring school wellness activities (Koriakin et al., 2020; Austin et al., 2006). Assessments should be conducted regularly and should inform quality improvement plans tailored to the specific school context, SMART (specific, measurable, achievable, relevant, time-bound) goals, parent and community involvement, and data-driven decisions and monitoring (Carvalho et al., 2022).

Penn State PRO Wellness, within Penn State College of Medicine's Department of Pediatrics, supports community-based research and educational programming in school settings. Launched in 2013, the PRO Wellness Healthy Champions program offers K-12 schools in Pennsylvania a free web-based assessment to measure their health promotion efforts (Francis et al., 2019). Schools completing the assessment, thereby enrolling in the Healthy Champions program, receive a custom report describing the results, areas for improvement, and access to supporting resources. Further program details are outlined in previous publications (Francis et al., 2019).

The Healthy Champions assessment was adapted from the CDC's SHI, a comprehensive tool for evaluating school health practices (Francis et al., 2019). While the SHI is a guiding tool, it requires significant time, personnel investment, and ideally outside facilitation (Austin et al., 2006; Brener et al., 2025). The Healthy Champions assessment was designed to mitigate the barriers preventing Pennsylvania schools from completing evaluation, such as allowing a single school representative with minimal support from additional staff, thus making the assessment time efficient (Francis et al., 2019). The resulting tailored report provides actionable recommendations to improve the school's health environment. Prior data collected demonstrated that schools enrolled annually saw significant improvement in their wellness scores (Francis et al., 2019).

Evolving best practices, new research, and shifting national school health priorities necessitated revision of the Healthy Champions assessment for the 2024–2025 program year. This manuscript describes the updated assessment and outcomes from participating Pennsylvanian schools.

## 2 Methods

### 2.1 Study design and population

Since its inception, the Healthy Champions assessment has aligned with recognized school health models, including the Coordinated School Health Model and its evolution to the WSCC model. Previous versions of the assessment (Francis et al., 2019; Hoke et al., 2024a) were adapted from the CDC's SHI and provided Pennsylvania schools with the ability to quickly assess and consider solutions to improve their school wellness environments with limited financial resources or human capital.

Revisions began with a literature review on recently updated WSCC-aligned best practices and policies to identify key factors impacting school health (Hoke et al., 2024b; Cohen et al., 2021; Flure et al., 2020; Michael et al., 2021; McGorry and Mei, 2018; Shelton and Owens, 2021). The findings informed updates and reorganization into five sections: (1) Physical Education and Activity, (2) Nutritional Environment, (3) Counseling and Psychological Services, (4) Health Services and Education, and (5) Health Environment and Culture. Each section included six multiple-choice questions with three response options. The response options allow schools to select a choice that either exceeds, meets, or does not meet best practices or regulatory standards. In addition to measuring school wellness practices, the choice selection allows schools to clearly see what practices can be employed to ensure best practices or regulatory standards are met.

The updates expanded the assessment beyond its original focus on nutrition and physical activity to include mental health and services provided by school nurses. This adjustment was well aligned with revised focus areas following the COVID-19 pandemic and the resulting changes in adolescent school experience (Next, 2021). New questions also addressed employee wellness (CDC - Whole School, 2026; Research, 2020).

The new assessment was pilot tested in January 2024 with four school professionals (two school nurses, one physical education and health teacher, and one administrator). Participants completed the assessment and evaluated the clarity of the instructions and questions, response choices, and ability to answer questions independently. Feedback resulted in expanded instructions, updated response choices, and confirmation that respondents could complete the assessment with minimal school staff collaboration.

Penn State PRO Wellness offers the Healthy Champions assessment to all K-12 schools in Pennsylvania. One school representative gathers information on school health practices collaborating minimally with other school staff, and submits the web-based assessment. It is promoted via Penn State PRO Wellness' digital newsletter, website, and through the Pennsylvania Department of Education, Department of Health's school nurse division, and the Pennsylvania Association of School Nurses and Practitioners. Enrollment for the 2024–2025 program was open from April–June 2024.

The Human Subjects Protection Office determined the activity does not meet the definition of human subjects research; therefore, Institutional Review Board (IRB) review is not required.

### 2.2 Measures

Data was collected using REDCap (Research Electronic Data Capture) hosted at Penn State College of Medicine (Harris et al., 2009). Along with standard demographic data, the assessment included five sections, each with six multiple-choice questions and one open-ended question for schools to report section-specific successes and challenges (Appendix A). Where relevant, questions within each section also addressed professional development for employees involved in school health and employee wellness. While these responses contributed to score calculations, specific outcomes related to employee wellness and professional development are addressed in a companion publication (citation forthcoming).

Each enrolled school received a customized report based on assessment responses. The report highlighted strengths and areas for improvement and directed schools to resources that support wellness practices, using low-cost, low-staffing strategies, thereby helping schools improve their scores over time.

### 2.3 Statistical analysis

The data set was manually cleaned by the program team. Unfinished and duplicate submissions, identified by identical addresses and contact information, were removed, and the first record submitted was retained. As this program primarily serves Pennsylvania schools, out-of-state submissions were removed for this analysis.

In each section, the multiple-choice questions were scored 0–2 points based on whether the responses exceeded (2 points), met (1 point), or did not meet best practices or regulatory standards (0 points). This simplified scoring system is similar to our previously published assessment (Francis et al., 2019). Open-ended questions were unscored.

Section scores were calculated by averaging the six scored questions, yielding an average score between 0 and 2. Average scores were grouped into three ranges, indicating their adherence to best practices (Table 1).

**Table 1:** Score ranges applied to each assessment section for the 2024–2025 Healthy Champions assessment.

Score Range	Explanation
1.34–2	Section scores indicate advanced achievement of programs, policies, or initiatives that exceed best practice or regulatory standards.
0.67–1.33	Section scores generally meet best practices or regulatory standards and trend toward advanced programming in this area.
0–0.66	Section scores identify room for improvement to meet best practices or regulatory standards in this area.

All data were summarized with mean scores and standard deviation by section, question, and section by respondent role type. To explore any associations between the section scores and affiliated respondent role type (i.e., Physical Education and Activity and Health or Physical Education Teacher), an analysis of variance was performed. *P*-values and confidence limits for comparisons made between respondent role types were adjusted for multiple comparisons using the Tukey-Kramer method. All statistical analyses we performed with a 0.05 level of significance using SAS version 9.4 (SAS Institute, Cary, NC).

## 3 Results

### 3.1 Demographics

In the 2024–2025 program, 328 Pennsylvania schools enrolled across 54 out of 67 Pennsylvania counties (80.6%). School types include public (*n* = 291, 88.7%), private (*n* = 19, 5.8%), and charter (*n* = 18, 5.5%) schools. Responding school professionals included school nurses (*n* = 237, 72.3%), health or physical education teachers (*n* = 34, 10.4%), administrators (*n* = 32, 9.8%), food service professionals (n = 18, 5.5%), classroom teachers (*n* = 4, 1.2%), and other school professionals (n = 3, 0.9%). Two-thirds of schools were urban (*n* = 221, 67.4%), based on classification from the Center for Rural Pennsylvania (Rural Urban Definitions, 2025).

### 3.2 Overall scores

Individual section scores were calculated based on each section's six questions (Table 2). The School Health Environment and Culture section had the lowest mean score at 1.06, and nearly one quarter (*n* = 81, 24.7%) of scores fell into the “not meeting best practices/standards” range (hereafter, standards). Health Services and Education had the greatest mean section score at 1.41, in addition to the lowest number of schools scoring in the “not meeting standards” range (*n* = 9, 2.7%). Two-thirds (*n* = 219, 65.2%) of schools reported meeting or exceeding standards in all five sections, 80 (23.8%) schools reported meeting or exceeding standards in four sections, and 37 (11%) schools reported meeting or exceeding standards in three or fewer sections.

**Table 2:** Healthy Champions assessment section scores in Pennsylvania schools from April to June 2024.

Section	Total Count	Not Meeting Best Practices and Regulatory Standards 0.0–0.66 (%)	Meeting Best Practices and Regulatory Standards 0.67–1.33 (%)	Exceeding Best Practices and Regulatory Standards 1.34–2 (%)	Mean Score (SD)
Physical Education and Activity	328	32 (9.7%)	177 (54.0%)	119 (36.3%)	1.19 (0.48)
Nutritional Environment	⁎322	16 (5.0%)	179 (55.6%)	127 (39.4%)	1.30 (0.43)
Counseling and Psychological Services	328	22 (6.7%)	142 (43.3%)	164 (50.0%)	1.34 (0.44)
Health Services and Education	328	9 (2.7%)	138 (42.1%)	181 (55.2%)	1.41 (0.38)
School Health Environment and Culture	328	81 (24.7%)	154 (46.9.0%)	93 (28.4%)	1.06 (0.54)

⁎ Schools who do not offer a school meal program do not receive a nutritional environment section score.

### 3.3 Wellness efforts and scores by section

Physical Education and Activity. 90% of schools reported meeting (*n* = 170) or exceeding (*n* = 123) standards in physical education programming (Table 3). Open-ended question responses included funding and staffing cuts resulting in decreased opportunities for physical activity and constraints regarding physical education instruction.

**Table 3:** Healthy Champions student focused assessment questions scores from Pennsylvania schools from April to June 2024.

1. Physical Education and Activity, *n* = 328				
Question⁎		**Does not meet best practices or regulatory standards** **(%)**	**Meets best practices or regulatory standards** **(%)**	**Exceeds best practices or regulatory standards** **(%)**
*1.1*	Please describe your school's role in helping students reach 20 min of physical activity each school day.	35 (10.7%)	56 (17.1%)	237 (72.2%)
*1.2*	Please describe your school's physical education program.	35 (10.7%)	170 (51.8%)	123 (37.5%)
*1.3*	Please describe your school's support of before/after school physical activity.	97 (29.6%)	75 (22.8%)	156 (47.6%)
*1.4*	Please describe how your school allows families/community members to utilize recreational equipment and facilities.	64 (19.5%)	122 (37.2%)	142 (43.3%)
2. Nutritional Environment, *n* = 322				
Question		**Does not meet best practices or regulatory standards**	**Meets best practices or regulatory standards**	**Exceeds best practices or regulatory standards**
*2.1*	Ensuring adequate time for students to eat can improve meal consumption. Which of the following options best describes your school?	4 (1.2%)	58 (18.0%)	260 (80.8%)
*2.2*	Please describe the practices used by your school meal program.	63 (19.6%)	142 (44.1%)	117 (36.3%)
*2.3*	Please describe your school's practices related to establishing nutrition standards for foods and beverages offered to students outside of the school meal program	86 (26.7%)	119 (37.0%)	117 (36.3%)
*2.4*	Please describe your school's practices related to student access to drinking water.	0 (0.0%)	40 (12.4%)	282 (87.6%)
3. Counseling and Psychological Services, n = 328				
Question		**Does not meet best practices or regulatory standards**	**Meets best practices or regulatory standards**	**Exceeds best practices or regulatory standards**
*3.1*	Please describe your school's prevention-based activities/curriculum to students.	51 (15.5%)	138 (42.1%)	139 (42.4%)
*3.2*	Please describe your school's universal mental health screenings.	46 (14.0%)	207 (63.1%)	75 (22.9%)
*3.3*	Please describe your school's resources and strategies for early interventions.	18 (5.5%)	105 (32.0%)	205 (62.5%)
*3.4*	Please describe your school's psychological counseling services by a licensed mental health professional.	5 (1.5%)	131 (40.0%)	192 (58.5%)
4. Health Services and Education, n = 328				
Question		**Does not meet best practices or regulatory standards**	**Meets best practices or regulatory standards**	**Exceeds best practices or regulatory standards**
*4.1*	Please describe your school's Certified School Nurse (CSN) to student ratio.	6 (1.9%)	192 (58.5%)	130 (39.6%)
*4.2*	Please describe how your school shares BMI (Body Mass Index) screening results with parents.	37 (11.3%)	111 (33.8%)	180 (54.9%)
*4.3*	Please describe your school's support of health care for students.	34 (10.4%)	126 (38.4%)	168 (51.2%)
*4.4*	Please describe who health education is provided to your school.	35 (10.7%)	125 (38.1%)	168 (51.2%)
*4.5*	Please describe your school's promotion of health in daily practices.	44 (13.4%)	76 (23.2%)	208 (63.4%)
5. School Health Environment and Culture, n = 328				
Question		**Does not meet best practices or regulatory standards**	**Meets best practices or regulatory standards**	**Exceeds best practices or regulatory standards**
*5.1*	Please describe how often your school's health council (or equivalent) meets.	122 (37.2%)	136 (41.5%)	70 (21.3%)
*5.2*	Please describe the makeup of your health council.	125 (38.1%)	88 (26.8%)	115 (35.1%)
*5.3*	Please describe how your school involves families in reinforcing healthy behaviors.	63 (19.2%)	172 (52.4%)	93 (28.4%)
*5.4*	Please describe how often your school reviews and updates its local wellness policies.	82 (25.0%)	125 (38.1%)	121 (36.9%)
*5.5*	Please describe how your school monitors and evaluates the implementation of district health and wellness policies and programs.	118 (36.0%)	125 (38.1%)	85 (25.9%)

⁎ Questions in this table relate to practices supporting student wellness. Questions relating to employee wellness and professional development are included in a companion publication.

Nutritional Environment. 98.8% of schools reported meeting (*n* = 58) or exceeding (*n* = 260) standards related to adequate time for students to eat meals. However, 27% (*n* = 86) of schools reported not meeting standards for practices related to foods and beverages offered to students outside of the school meal program (Table 3). Open-ended responses indicated both challenges and successes related to the school meal program. Common themes included universal free breakfast and lunch and concerns about providing overly processed foods high in fat and sugar content.

Counseling and Psychological Services. 99% of schools reported meeting (*n* = 131) or exceeding standards (*n* = 192; Table 3) related to the provision of psychological counseling services Open-ended responses highlighted schools' use of Student Assistance Programs (SAP) and partnerships with local external counseling services to meet the needs of student populations.

Health Services and Education. 99% of schools reported meeting (n = 192) or exceeding (*n* = 130) standards for the Pennsylvania Certified School Nurse to student ratio. Schools highlighting challenges relating to the Pennsylvania Certified School Nurse to student ratio in the open-ended question. One nurse explained, “More nursing staff would help to be able to do more health promotion activities,” and “We have one school nurse for 2 buildings that are miles apart with many chronic conditions.”

School Health Environment and Culture. Nearly 40% of schools reported not meeting standards for school health councils, including frequency of meetings (*n* = 122, 37.2%) and composition of the council (*n* = 125, 38.1%; Table 3). Similarly, 36% of schools were not meeting standards for monitoring and evaluating implementation of health and wellness policies and programs (*n* = 118). Open-ended responses highlighted barriers to implementing or sustaining a wellness council and wellness policies, citing “turnover in administration” and focus on “education over health”.

### 3.4 Section scores and respondent role type

Mean section scores by respondent role type (Table 4) and comparison of section scores by respondent role types (Table 5) were examined for potential differences. Food services professionals scored higher than other roles across most sections.

**Table 4:** Mean section scores in the Healthy Champions assessment by respondent role type in Pennsylvania schools from April to June 2024.

	Section Mean (SD)				
Role Type	Physical Education and Activity	Nutritional Environment	Counseling and Psychological Services	Health Services and Education	School Health Environment and Culture
School Nurse, n = 237	1.19 (0.48)	1.27 (0.44)	1.34 (0.42)	1.40 (0.37)	1.03 (0.56)
Health/Physical Education Teacher, n = 34	1.08 (0.46)	1.24 (0.42)	1.30 (0.44)	1.37 (0.38)	1.06 (0.52)
Food service Professional, n = 18	1.55 (0.38)	1.72 (0.21)	1.78 (0.22)	1.70 (0.28)	1.20 (0.46)
Administration; n = 32	1.21 (0.42)	1.47 (0.26)	1.28 (0.49)	1.50 (0.36)	1.20 (0.46)
Other (Classroom Teachers and Self-Described Others); *n* = 7	0.81 (0.42)	0.95 (0.27)	1.02 (0.43)	1.10 (0.38)	0.76 (0.62)

**Table 5:** Comparisons of mean section scores in the Healthy Champions assessment by respondent role types in Pennsylvania Schools from April to June 2024.

		Difference of means and comparison p-value⁎				
Role Type 1	Role Type 2	Physical Education and Activity	Nutritional Environment	Counseling and Psychological Services	Health Services and Education	School Health Environment and Culture
Administration	Food Service Professional	−0.34, *p* = 0.11	−0.25, *p* = 0.24	−0.50, *p* < 0.00	−0.21, *p* = 0.31	0.00, *p* = 1.00
Administration	Health and/or Physical Education Teacher	0.13, *p* = 0.82	0.24, *p* = 0.14	−0.02, p = 1.00	0.13, *p* = 0.63	0.14, *p* = 0.84
Administration	Other (Classroom Teachers and Self-Described Others	0.40, *p* = 0.25	0.52, *p* = 0.02	0.25, *p* = 0.61	0.40, *p* = 0.08	0.44, *p* = 0.29
Administration	School Nurse	0.02, p = 1.00	0.21, *p* = 0.06	−0.06, *p* = 0.94	0.10, p = 0.61	0.17, *p* = 0.46
Food Service Professional	Health and/or Physical Education teacher	0.46, *p* = 0.01	0.49, p < 0.00	0.48, *p* = 0.00	0.34, p = 0.02	0.14, *p* = 0.90
Food Service Professional	Other (Classroom Teachers and Self-Described Others	0.74, p = 0.00	0.77, p < 0.00	0.75, p < 0.00	0.61, p = 0.00	0.44, *p* = 0.36
Food Service Professional	School Nurse	0.35, p = 0.02	0.46, p < 0.00	0.44, p < 0.00	0.31, p = 0.01	0.17, *p* = 0.70
Health and/or Physical Education teacher	Other (Classroom Teachers and Self-Described Others	0.27, p = 0.63	0.29, *p* = 0.45	0.28, *p* = 0.52	0.27, *p* = 0.40	0.30, p = 0.70
Health and/or Physical Education teacher	School Nurse	−0.11, *p* = 0.71	−0.03, p = 1.00	−0.04, *p* = 0.99	−0.03, p = 0.99	0.03, *p* = 1.00
Other (Classroom Teachers and Self-Described Others	School Nurse	−0.38, *p* = 0.21	−0.31, *p* = 0.27	−0.31, *p* = 0.30	−0.30, *p* = 0.22	−0.27, *p* = 0.69

⁎ p-values calculated using the section mean scores reported in Table 4.

When comparing respondent role types with their associated section score, Nutritional Environment, as reported by Food Service professionals, was the only association that showed significantly higher mean score than all other respondent types (Table 5). No other role type and section association showed significant statistical difference.

## 4 Discussion

The 2024–2025 Healthy Champions assessment offers insight into how Pennsylvania schools support health. Most schools reported meeting standards, evidenced by 89% of schools that achieving this score range in four or more sections. Mean scores in three sections met standards, with the remaining two sections exceeding standards.

Results varied across sections, with higher compliance in sections more regulated or supported by state or national policies, such as Health Services and Education, Nutritional Environment, and Counseling and Psychological Services. For example, 73% of schools reported meeting or exceeding standards related to school meal nutritional standards. These standards are a requirement for schools participating in the Free and Reduced Cost Meal Program, which provides subsidies to schools to support students' access to nutritious food. In contrast, over one-third of schools did not meet standards in wellness policy or program evaluation which is not mandated or regulated by Pennsylvania or national-level policies. This highlights the importance of state and national policy in ensuring schools meet standards. Additional strengths and areas for improvement are further outlined below.

Reported successes relate to practices in the Counseling and Psychological Services section. School attention on student mental health has gained significant support since the COVID-19 pandemic (Walters, 2021). Specifically, infrastructure has been developed by the Pennsylvania Department of Education and Office of Mental Health and Substance Abuse Services, making the commonwealth well-established as a leader in student assistance programming (Fertman et al., 2001; Sekhar et al., 2023). Healthy Champions schools reported practices that generally met or exceeded standards, with over 85% (*n* = 282) conducting health screenings, and nearly 95% of schools (*n* = 310) utilizing a variety of best practice early intervention efforts. These data are comparable to the 78% of schools, Nationally, practicing mental health screenings to identify students in need of services and 92% of schools providing universal mental health promotion programs, as reported by CDC's School Health Profiles (CDC School health profiles, 2024a) However, opportunities exist to improve resources to support student mental health, as data from the Pennsylvania Youth Survey still indicate 16.1% of students have seriously considered suicide and 12.5% planned suicide in 2023 (*Pennsylvania Youth Survey (PAYS). Commonwealth of Pennsylvania. Accessed July 28, 2025. https://www.pa.gov/agencies/pccd/programs-and-services/juvenile-justice-and-delinquency-prevention/pays#accordion-3eefc3bc45-item-3f209d*2872, 2023). As only one quarter of Healthy Champions schools reported universal screening, expanding screening resources could strengthen early identification transitioning students to services (Sekhar et al., 2021).

Responding Pennsylvania schools also reported excellence in the provision of student access to drinking water and the time provided for meal consumption. These basic practices should remain the goal; however, future resource efforts for improving school health should be redirected to areas in greater need of improvement.

While most schools (*n* = 320, 98.2%) indicated meeting the Pennsylvania requirements for a school nurse to student ratio of 1:1500 (*Act 14 Chapter 14 Section 2 - The Official Website of the Pennsylvania General Assembly. Published March* 10, 1949. Accessed July 28, 2025. https://www.palegis.us/statutes/unconsolidated/law-information/view-statute?txtType=PDF&SessYr=1949&ActNum=0014.&SessInd=0. https://www.palegis.us/statutes/unconsolidated/law-information/view-statute?SESSYR=1949&SESSIND=0&ACTNUM=14&SMTHLWIND=&CHPT=14&SCTN=2&SUBSCTN, 1949), respondents expressed concerns about the adequacy of this standard, the burden placed on school nurses, and potential implications for student safety. These concerns align with nationwide discussions citing the need to reassess mechanisms used to determine the number of full-time equivalent school nurse positions needed to best support the school-aged populations (Cogan, 2024). The National Association of School Nurses 2020 position statement identified the need to revise the antiquated measure of school nurse success (National Association of school nurses (NASN) position statement: school nurse workload, 2025). Rather than a static nurse-to-student ratio, new recommendations include considering the complexity of a nurse's caseload when determining the number of Certified School Nurses (CSN) and support professionals needed to best support a student population (National Association of school nurses (NASN) position statement: school nurse workload, 2025). Studies suggest students benefit from lower ratios (Best et al., 2021), including increased capacity for care, increased nurse self-efficacy for care provided, and reduced absenteeism. Consideration should be given to both updating outdated Pennsylvania policies regarding CSN-to-student ratios and the improvement of school nurses' capacity to provide health services and serve as a leader in health promotion.

Schools in the 2024/25 Healthy Champions program were generally successful at supporting wellness in conventional spaces like the cafeteria and gymnasium. However, schools most commonly had areas for improvement in nonconventional “wellness” spaces, such as the classroom and within activities occurring out of school hours. This is evidenced by nearly one-third of schools failing to meet standards relating to physical activity before or after school (*n* = 97, 29.6%), allowing family and community members to access recreational equipment and facilities (*n* = 64, 19.5%), and establishing food standards for food served outside of the school meals program (*n* = 86, 26.7%). Data suggests schools fail to meet key components of both the WSCC model (Lewallen et al., 2015) and a Comprehensive School Physical Activity Program (CSPAP) (CDC - Whole School, 2026) which serve as the recognized frameworks for addressing school health and comprehensive physical activity in schools, respectively. Better alignment to these practices is necessary to achieve a wholistic student-centered approach to improving school wellness.

Of the observed weaknesses, the most attainable improvements remain in school practices related to School Health Environment and Culture. 25% of schools (*n* = 82) reported not reviewing local wellness policies triennially. On trend with national data reported in the CDC's School Health Profiles, schools report not measuring compliance with wellness policies (CDC School health profiles, 2024b). Additionally, schools reported poor utilization of wellness councils and failed to include diverse perspectives in their composition. Finally, significant opportunities exist to regularly monitor and evaluate wellness programs. Existing literature demonstrates that the successful implementation of those three activities (1) reviewing wellness policies, (2) regular meetings of a diverse wellness council, and (3) evaluation of health promotion programs is significantly associated with improved school wellness (Hoke et al., 2024b). Where some improvements to school wellness initiatives require a significant investment in infrastructure, staffing, or curriculum, improving activities related to policies, wellness councils, and program evaluation are low-cost solutions to hold schools accountable for their wellness environment success.

Although some comparisons between role types and section score were statistically significant, these data do not suggest an association between respondent role type and their associated section scores. Rationale for higher mean scores reported by food service professionals is unknown. The variation in mean scores observed by role type warrant additional examination of the impact of certain school professionals and their role as champions.

While the results do not indicate association, these findings underscore the importance of respondents collaborating with other school health professionals in the school to answer questions and cautiously interpreting results with consideration to contextual factors. Further validation is needed to determine whether these role-based variations reflect actual differences in practices or differences in how a respondent's role impacts their perception and therefore reporting of school health practices.

Although the Healthy Champions assessment is aligned with the WSCC model, the instrument has not undergone formal validation, therefore interpretations should be made cautiously. Furthermore, the data reflect a subset of self-selected Pennsylvania schools that voluntarily enroll and report practices, introducing the potential for self-selection bias and social desirability bias. Additionally, recruitment methods prevent accurate calculation of the exact number of unique school buildings reached (i.e., a denominator to determine survey response rate). Finally, because the assessment was tailored to Pennsylvania-specific practices and regulations, results may not be generalizable to other states or regions.

## 5 Conclusions

Results of the 2024–2025 Healthy Champions assessment reveal trends that warrant continued observation. While most schools meet or exceed standards, creating a comprehensive, student-centered school health environment remains a challenge. Some wellness practices are more attainable than others, highlighting a need to further examine factors that support or hinder comprehensive strategies. Results will guide the program team in developing wellness resources, therefore better supporting Pennsylvania schools in areas with the highest need for improvement. Both the Healthy Champions assessment and program will continue to serve as a practical tool for identifying gaps, providing data-driven feedback and connecting schools to low-cost, high-impact strategies. Continued annual participation and enrollment in the Healthy Champions program support holistic school health practices that promote both academic success and student well-being.

## CRediT authorship contribution statement

**Allison M. Linton:** Writing – review & editing, Writing – original draft, Project administration, Methodology, Formal analysis, Conceptualization. **Alicia M. Hoke:** Writing – review & editing, Writing – original draft, Supervision, Project administration, Methodology, Conceptualization. **Lindsay A. Aluquin:** Writing – review & editing, Formal analysis, Data curation. **Erik B. Lehman:** Writing – review & editing, Formal analysis. **Deepa L. Sekhar:** Writing – review & editing, Supervision, Funding acquisition, Conceptualization.

## Declaration of generative AI and AI-assisted technologies in the writing process

During the preparation of this work the authors used AI to proofread the text for grammar and spelling errors. After using this tool/service, the authors reviewed and edited the content as needed and take full responsibility for the content of the published article.

## Declaration of competing interest

The authors declare that they have no known competing financial interests or personal relationships that could have appeared to influence the work reported in this paper.

## Data Availability

The authors do not have permission to share data.

## Appendix A Supplementary data

Supplementary material. Image 1
