# Supplementary material for: Evaluating school health practices in Pennsylvania: Development, results, and insights of the revised 2024–2025 healthy champions assessment
Source: Prev Med Rep. 2026 Jan 14;62:103386. doi: 10.1016/j.pmedr.2026.103386 (PMC12860691; doi:10.1016/j.pmedr.2026.103386)
Supplement: Supplementary file 1 — Supplementary material. [file mmc1.docx]

**Appendix for:**

***Evaluating School Health Practices in Pennsylvania: Development, Results and Insights of the Revised 2024–2025 Healthy Champions Assessment***

**Appendix A**. Scored questions in the Healthy Champions assessment for Pennsylvania schools from April to June 2024.

| Section 1: Physical Education and Activity | | |
| --- | --- | --- |
| *Question* | | ***Response Choices*** |
| *1.1* | The comprehensive school physical activity program (CSPAP) framework can help schools identify activities that can improve opportunities for students to meet the recommended amount of physical activity each day. A CSPAP is comprised of 5 components – Physical education, physical activity during school, physical activity before and after school, staff involvement, and family and community involvement.^1,2^  Answer the following questions to describe your school’s physical activity environment. | 1. All students have adequate opportunity to achieve 20 minutes of physical activity during the school day AND this practice is clearly supported by school administration 2. All students have adequate opportunity to achieve 20 minutes of physical activity during the school day 3. Few students are provided opportunity to achieve 20 minutes of physical activity during the school day |
| *1.2* | Physical education is the cornerstone of a comprehensive school physical activity program. The four essential components of a quality physical education program include:   1. Policies and environment: schools define physical education expectations through specific physical education policy and policy guidance. 2. Curriculum: schools have a written, standards-based curriculum. 3. Appropriate instruction: physical education takes into account the diverse developmental levels that students in all grade levels will exhibit. 4. Student assessment: physical educators consider evidence of student achievement and make inferences about student progress based on that evidence.^1,2^   Please describe your school’s physical education program? | 1. Our school's physical education program meets all four essential components of a quality physical education program AND students meet the recommended weekly instructional minutes (150 minutes/week in elementary and 225 minutes/week in middle and high school). 2. Our school's physical education program meets all four essential components of a quality physical education program. 3. Our school's physical education program meets some, but not all, of the four essential components of a quality physical education program. |
| *1.3* | Providing opportunities for students to be active before or after school can replace periods of sedentary time and contribute to the 60 minutes of daily physical activity recommended for children and adolescents. Examples of physical activity before/after school include:   1. Walking/biking to/from school program 2. Interscholastic sports 3. Physical activity clubs and intramural programs (e.g., programs that are voluntary, student-centered, and give equal opportunity for all students to participate) 4. Physical activity in school-based childcare programs 5. Encourage informal recreation or play on school grounds 6. Encourage integrating physical activity in homework during out of school hours^3^   Please describe your school’s support of before/after school physical activity. | 1. Our school provides 3 or more of the opportunities listed AND this practice is clearly supported by the school administration. 2. Our school provides 3 or more of the opportunities listed. 3. Our school provides 2 or fewer of the opportunities listed |
| *1.4* | Engaging families and the community can maximize the use of resources (physical and human capital) and create a connection between school and community-based physical activity opportunities. This may include establishing joint- or shared-use agreements with schools or coordinating with community organizations to provide physical activity programs outside of school hours.^4^  Please describe how your school allows families/community members to utilize recreational equipment and facilities. | 1. Our school partners regularly with families/community members to create joint- or shared-use agreements for access to recreational equipment or facilities, regardless of the need for school resources (ex. Janitorial staff time). 2. Our school allows families/community members to access recreational equipment or facilities that do not require the school to allocate resources (ex. Janitorial staff time). 3. Our school does not allow families/community members to access any recreational equipment or facilities outside of school hours. |
| *1.5* | Please describe your school’s opportunities for staff to participate in training and professional development to improve/increase physical activity for students (e.g., providing training, financial support for outside training).^5^ | 1. Opportunities are available AND noticeably supported/encouraged by the school administration (I.e., financially, in performance reviews). 2. Opportunities are available on a case-by-case basis. 3. Opportunities for professional development of this kind are not generally requested or supported. |
| *1.6* | Providing employee wellness programming for staff can not only improve staff health but can result in staff modeling positive physical activity practices for their students. Best practices for providing physical activity opportunities for staff include:     1. Providing opportunities for staff to be physically active during the school day 2. Providing on-site physical activity opportunities (ex. group fitness classes led by staff member or community partner) 3. Covering fees for participating in group exercise and/or fitness centers through employee benefits package^6,7^   Please describe your school’s support of staff physical activity. | 1. Our school provides free or discounted access to physical activity through our employee benefits package AND provides opportunities at the school (before/during/after) to participate in physical activity. 2. Our school provides free or discounted access to physical activity through our employee benefits package. 3. Our school does not provide any benefits to staff related to physical activity. |
| *1.7* | Please share relevant challenges or successes regarding your school's physical education/activity environment.  *Note: This question will NOT be used to determine your Star Status. | |
| Section 2: Nutritional Environment* | |  |
| *2.1* | Ensuring adequate time for students to eat can improve meal consumption.^8,9^  Which of the following options best describes your school? | 1. Most students get 20 or more minutes of seated lunch time AND this practice is enforced by school administrators. 2. Most students get 20 or more minutes of seated lunch time. 3. Few students get 20 or more minutes of seated lunch time. |
| *2.2* | The following practices have been shown to improve the appeal/palatability of school meals and students’ dietary behaviors:   1. Taste tests 2. Incentives/rewards for trying new foods 3. Pre-slicing fruit 4. Rearranging the serving line 5. Using signage and prompts to encourage consumption of healthy choices 6. Changing portion sizes 7. Presenting foods in attractive ways 8. Using creative names for menu items^10^   Please describe the practices used by your school meal program. | 1. Our school uses 5 or more of these strategies 2. Our school uses 3-4 of these strategies 3. Our school uses 2 or fewer of these strategies |
| *2.3* | Please describe your school’s practices related to establishing nutrition standards for foods and beverages offered to students outside of the school meal program (I.e., celebrations and rewards).^11,12^ | 1. Our school has established nutrition standards for foods provided outside of the school meal program AND these are enforced in a standard way by school administrators. 2. Our school has established nutrition standards for foods provided outside of the school meal program. 3. Our school does not have established nutrition standards for foods provided outside of the school meal program. |
| *2.4* | Please describe your school’s practices related to student access to drinking water.^13^ | 1. Students have access to free, clean drinking water through water fountains or bottle fillers AND can access a drink of water at any point during the school day (I.e., water bottles at their seat). 2. Students have access to free, clean drinking water through water fountains or bottle fillers. 3. Students do not have regular access to free, clean drinking water throughout the school day. |
| *2.5* | Please describe the professional development practices of your school nutrition professional(s).^14^ | 1. Professional development for school nutrition professionals occurs at least annually AND is noticeably supported/encouraged by school administration (I.e., financially, in performance reviews) 2. Professional development for school nutrition professionals occurs at least annually. 3. Professional development for school nutrition professionals occurs less than annually, or infrequently. |
| *2.6* | Providing employee wellness programming for staff can improve staff health and result in staff modeling positive healthy eating practices for their students. Best practices for providing a positive nutritional environment for staff include:   1. Providing educational materials (i.e., brochures, posters, newsletters, videos) that address healthy eating 2. Providing free or subsidized lifestyle coaching/counseling or self-management programs that equip employees with skills and motivation to set and meet their personal nutritional goals (i.e., healthy eating and budgeting, chronic disease management through diet, dietitian counseling) 3. Providing free or subsidized participation in nutrition programs (i.e., WeightWatchers)^6,15^   Please describe your school’s support of staff nutrition. | 1. Our school provides free or discounted access to nutrition/healthy eating education or programs through our employee benefits package AND provides healthy food and beverage options at employee celebrations and gatherings. 2. Our school provides free or discounted access to nutrition/healthy eating education or programs through our employee benefits package. 3. Our school does not provide any benefits for staff related to nutrition or healthy eating. |
| *2.7* | Please share relevant challenges or successes regarding your school's nutritional environment.  *Note: This question will NOT be used to determine your Star Status. | |
| Section 3: Counseling and Psychological Services | |  |
| *3.1* | Please describe your school’s prevention-based activities/curriculum to students? (I.e., seminars, health education curriculum, mental health week, information sent home).^16^ | 1. All students participate in prevention-based activities, education, or receive educational resources AND noticeably supported by administration (I.e., financially supported, etc.) 2. All students participate in prevention-based activities, education, or receive educational resources. 3. These resources and education are only provided because of an incident or not at all. |
| *3.2* | Please describe your school’s universal mental health screenings.^17,18^ | 1. Our school conducts mental health screenings for all students at regular intervals (i.e. annually or bi-annually) 2. Our school conducts mental health screenings for students only when prompted by an incident, upon request or referral (i.e. Student Assistance Program). 3. Our school does not conduct mental health screenings. |
| *3.3* | Mental health early intervention is recommended for students exhibiting risk factors associated with potential issues that are not fully manifested. Early intervention can positively affect the mental health of at-risk students. Examples of early intervention in schools include:   1. Small group interventions for students with similar needs 2. Motivational interviewing 3. Problem Solving Coaching 4. Mentoring 5. Classroom-based support (I.e., daily report cards, daily teacher check-ins, home-note systems)^19^   Please describe your school’s resources and strategies for early interventions. | 1. Our school provides 3 or more of the opportunities listed for mental health early intervention. 2. Our school provides at least 2 of these opportunities for mental health early intervention. 3. Our school does not use early intervention techniques. |
| *3.4* | Please describe your school's psychological counseling services by a licensed mental health professional (i.e., therapist, licensed counselor, psychologist, or social worker).^20^ | 1. These services are available to all students. 2. These services are available to high risk or referred students. 3. These services are not available at our school. |
| *3.5* | Please describe your school’s required mental health or suicide prevention training for school professionals (i.e., classroom teachers, support staff, food service staff, bus drivers).^21^ | 1. All school professionals (i.e., classroom teachers, support staff, food service staff, bus drivers) who interact with students receive training. 2. Some non-licensed professionals (i.e., classroom teachers, support staff, food service staff, bus drivers) receive training. 3. Training is only required for licensed professionals (i.e., counselors). |
| *3.6* | The CDC recommends practices to help workplaces reduce stress and depression for employees. Examples of these strategies that employers can implement includes:   1. Provide educational materials on stress management and preventing, detecting, and treating depression (i.e., brochures, videos, posters, newsletters). 2. Provide dedicated space that is quiet where employees can engage in relaxation activities such as deep breathing. 3. Sponsor or organize social activities designed to improve engagement with others for social support (I.e. team building, holiday parties, team picnics). 4. Conduct employee needs assessments.^6^   Please describe your school's mental health resources for staff. | 1. Our school utilizes 3 or more of the strategies listed. 2. Our school provides 1-2 of the strategies listed. 3. Our school does not provide resources to support staff mental health. |
| *3.7* | Please share relevant challenges or successes regarding your school's counseling and psychological services environment.  *Note: This question will NOT be used to determine your Star Status. | |
| Section 4: Health Services and Education | |  |
| *4.1* | Please describe your school’s Certified School Nurse (CSN) to student ratio.^22,23^ | 1. Our school has 2 or more Nurses (CSNs) for every 1500 students 2. Our school has 1 Nurse (CSN) for every 1500 students 3. Our school has 1 Nurse (CSN) for more than 1500 students, or no school nursing services |
| *4.2* | Please describe how your school shares BMI (Body Mass Index) screening results with parents.^24^ | 1. Communication includes direct methods (i.e., PRO Wellness BMI letter, email) and may also include indirect methods. 2. Communication includes indirect communication only (i.e., loaded in parent portal with no direct notification). 3. We do not share BMI screening results with parents. |
| *4.3* | Please describe your school's support of health care for students.^25^ | 1. Our school conducts advanced coordination of care and provides additional community-based health services for students (I.e., immunization clinic, dentistry services, mobile vision clinic, school-based health center). 2. Our school conducts advanced coordination of care for students by utilizing interdisciplinary school-based teams (I.e., certified school nurse, counselors, teachers) 3. Our school conducts basic coordination of care for students with chronic conditions. |
| *4.4* | Please describe who health education is provided to in your school.^26^ | 1. Standards-based health education is provided as a routine part of student curricula and is provided to all students (I.e., every grade). 2. Standards-based health education is provided as a routine part of curricula, but not to all students (I.e., only some grades). 3. Standards-based health education is not provided as a routine part of curricula (I.e., sporadic, or only a few topics covered). |
| *4.5* | Please describe your school’s promotion of health in daily practices? Healthy practices may include things like hand washing before lunch, physical activity breaks, mindfulness moments, positive morning announcements, etc.^27^ | 1. Healthy behaviors are promoted through classroom activities and daily routines AND this practice is clearly supported/encouraged by school administration. 2. Healthy behaviors are promoted through classroom activities and daily routines. 3. Healthy behaviors are minimally promoted (I.e., posters/signs) but not actively practiced. |
| *4.6* | Professional development support for nurses can include:   1. Funding support to join nursing associations 2. Funding support to travel to conferences 3. Funding support for professional development 4. Opportunities for earning continuing education credits^28^   Please describe your school's support of professional development for School Nurses. | 1. Opportunities are available AND noticeably supported/encouraged by school administration (I.e., in performance reviews). 2. Opportunities are available on a case-by-case basis. 3. Opportunities for professional development of this kind are not generally requested or supported. |
| *4.7* | Please share relevant challenges or successes regarding your school's health services and education environment.  *Note: This question will NOT be used to determine your Star Status. | |
| Section 5: School Health Environment and Culture | |  |
| *5.1* | Please describe how often your school’s health council meets.^29^ | 1. Our school health council meets at least four times per year. 2. Our school health council meets 1-3 times per year. 3. Our school does not have a school health council. |
| *5.2* | It is recommended that school health councils be comprised of diverse community stakeholders, including:   1. Parents 2. Students 3. Community Representatives 4. School administrators 5. Physical education teachers 6. Cafeteria managers 7. School nurses^29^   Please describe the makeup of your health council. | 1. Our school’s health council consists of 4 or more of the listed stakeholders. 2. Our school’s health council consists of 1-3 of the listed stakeholders. 3. Our school does not have a school health council. |
| *5.3* | Please describe how your school involves families in reinforcing healthy behaviors.^1,28^ | 1. Our school helps families reinforce healthy behaviors by providing information about health activities/resources (I.e., newsletters, social media channels, school websites) happening in the school AND invites families to participate in these school activities. 2. Our school helps families reinforce healthy behaviors by providing information about health activities/resources happening in the school (I.e., newsletters, social media channels, school websites). 3. Our school does not involve families in reinforcing healthy behaviors. |
| *5.4* | Please describe how often your school reviews and updates its local wellness policies.^29,30,31^ | 1. Our school reviews its local wellness policies at least annually. 2. Our school reviews its local wellness policies every 2-3 Years 3. Our school reviews its local wellness policies less often than every 3 years. |
| *5.5* | Please describe how your school monitors and evaluates the implementation of district health and wellness policies and programs.^29,30,31^ | 1. Our school sets goals and evaluates most, or all our health promotion programs AND this practice is clearly supported/encouraged by school administration. 2. Our school sets goals and evaluate most or all our health promotion programs. 3. Our school generally does not set program goals and evaluate our health promotion programs. |
| *5.6* | Please describe how your school educates employees about preventive services and benefits covered by their health insurance plan on an ongoing basis, above and beyond what occurs during annual health insurance enrollment (benefits could include smoking cessation medication and counseling, weight management tools, flu vaccinations, individualized health assessments, etc.).^6^ | 1. Our school educates employees on benefits that are covered by their health insurance plans throughout the year. 2. Our school educates employees only during health insurance enrollment. 3. Our school does not educate employees on benefits that are covered under insurance. |
| *5.7* | Please share relevant challenges or successes regarding your school's health environment and culture.  *Note: This question will NOT be used to determine your Star Status | |

Appendix A References

1. CDC - Whole School, Whole Community, Whole Child (WSCC). Centers for Disease Control and Prevention. June 26, 2026. Accessed July 28, 2025. https://www.cdc.gov/whole-school-community-child/about/index.html.
2. Michael SL, Wright C, Mays Woods A, et al. Rationale for the Essential Components of Physical Education. *Res Q Exerc Sport*. 2021;92(2):202-208. doi:10.1080/02701367.2020.1854427
3. Webster CA. The Comprehensive School Physical Activity Program: An Invited Review. *Am J Lifestyle Med*. 2022;17(6):762-774. Published May 25, 2022. doi:10.1177/15598276221093543
4. Dauenhauer B, Hodges Kulinna P, Marttinen R, Babkes Stellino M. Before- and After- School Physcial Activity: Programs and Best Practices. *J Phys Educ Recreat Dance*. June 2022. 93(5):20-26. doi:10.1080/07303084.2022.2053474
5. Promoting Physical Activity through the Shared Use of Community Recreational Resources. Active Living Research. April 2012. Accessed October 28, 2025. https://activelivingresearch.org/sites/activelivingresearch.org/file/ALR_Brief_SharedUse_April2012.pdf.
6. Ennis CD. Reimagining professional competence in physical education. *Motriz.* 2013;19(4):662-672. doi:10.1590/s1980-65742013000400001
7. CDC - Worksite Health ScoreCard. Centers for Disease Control and Prevention. Published online July 8, 2024. Accessed October 28, 2025. https://www.cdc.gov/workplace-health-promotion/php/scorecard/index.html.
8. Harris AR, Jennings PA, Katz D.A. et al*.* Promoting Stress Management and Wellbeing in Educators: Feasibility and Efficacy of a School-Based Yoga and Mindfulness Intervention. *Mindfulness.* 7, 143–154 (2016). https://doi.org/10.1007/s12671-015-0451-2
9. CDC School Nutrition – Time for Lunch. Centers for Disease Control and Prevention. Published online July 23, 2024. Accessed October 28, 2025. https://www.cdc.gov/school-nutrition/school-meals/time-for-lunch.html.
10. Cohen JFW, Hecht AA, Hager ER, Turner L, Burkholder K, Schwartz MB. Strategies to improve school meal consumption: A systematic review. *Nutrients*. 2021;13(10):3520. Published online October 7, 2021. doi:10.3390/NU13103520
11. Hildebrand D, Millburg Ely C, Betts NM. Time To Eat School Lunch Affects Elementary Students’ Nutrient Consumption. School Nutrition Association. Published online Fall 2018.42 (2). Accessed October 28, 2025. https://schoolnutrition.org/journal/fall-2018-time-to-eat-school-lunch-affects-elementary-students-nutrient-consumption/.
12. A Guide to Smart Snacks in School. United States Department of Agriculture – Food and Nutrition Service. Published online May 2022. Accessed October 28, 2025. https://www.fns.usda.gov/tn/guide-smart-snacks-school.
13. Rosenkranz R, Warner N, Yarrow L, Rosenkranz S. Use of Food Rewards in Education: Time to De-implement this Practice? School Nutrition Association. Published online Fall 2021.45 (2). Accessed October 28, 2025. https://schoolnutrition.org/journal/fall-2021-use-of-food-rewards-in-education-time-to-de-implement-this-practice/#full-article
14. CDC School Nutrition – Water Access in Schools. Centers for Disease Control and Prevention. Published online July 23, 2025. Accessed October 28, 2025. https://www.cdc.gov/school-nutrition/water-access/index.html.
15. Professional Standards for School Nutrition Professionals. United States Department of Agriculture – Food and Nutrition Service. Updated July 29, 2025. Accessed October 28, 2025. https://www.fns.usda.gov/tn/professional-standards.
16. Grimani A, Aboagye E, Kwak L. The effectiveness of workplace nutrition and physical activity interventions in improving productivity, work performance and workability: a systematic review. *BMC Public Health*. 2019;19(1):1676. Published 2019 Dec 12. doi:10.1186/s12889-019-8033-1
17. Mental Health Action Guide: Promoting Mental Health and Wellbeing in Schools. Centers for Disease Control and Prevention. Published online December 3, 2024. Accessed October 28, 2025. https://www.cdc.gov/mental-health-action-guide/about/index.html.
18. Siceloff ER, Bradley WJ, Flory K. Universal Behavioral/Emotional Health Screening in Schools: Overview and Feasibility. *Rep Emot Behav Disord Youth*. 2017;17(2):32-38.
19. McGorry PD, Mei C. Early intervention in youth mental health: progress and future directions. *Evid Based Ment Health*. 2018;21(4):182-184. doi:10.1136/ebmental-2018-300060
20. Dowdy E, Ritchey K, Kamphaus RW. School-Based Screening: A Population-Based Approach to Inform and Monitor Children's Mental Health Needs. *School Ment Health*. 2010;2(4):166-176. doi:10.1007/s12310-010-9036-3
21. Lenares-Solomon D, Brown MH, Czerepak R. The Necessity for School-Based Mental Health Services. *J Prof Couns Pract Theory Res.* November 4, 2019. 46(1–2). doi:10.1080/15566382.2019.1674074
22. 1949 Act 14 Chapter 14 Section 2 - The Official Website of the Pennsylvania General Assembly. Published March 10, 1949. Accessed July 28, 2025. https://www.palegis.us/statutes/unconsolidated/law-information/view-statute?txtType=PDF&SessYr=1949&ActNum=0014.&SessInd=0. https://www.palegis.us/statutes/unconsolidated/law-information/view-statute?SESSYR=1949&SESSIND=0&ACTNUM=14&SMTHLWIND=&CHPT=14&SCTN=2&SUBSCTN
23. National Association of School Nurses (NASN) Position Statement: School Nurse Workload – Essential Staffing for Safe and Equitable Student Care. *J Sch Nurs.* 2025;41(5):642-645. doi:10.1177/10598405251360761
24. Skantze C, Almqvist-Tangen G, Karlsson S. School nurses' experience of communicating growth data and weight development to parents of children 8 and 10 years of age. *BMC Public Health*. 2023;23(1):21. January 4, 2023. doi:10.1186/s12889-022-14941-9
25. CDC Managing Health Conditions in School – School Health Services. Centers for Disease Control and Prevention. Published online July 5, 2024. Accessed October 28, 2025. https://www.cdc.gov/school-health-conditions/health-services/index.html.
26. National Health Education Standards: Model Guidance for Curriculum and Instruction (3^rd^ Edition). 2022. Accessed October 28, 2025. https://www.schoolhealtheducation.org/wp-content/uploads/2024/02/National_Health_Education_Standards_Guide-02.21.2024.pdf
27. Pulimeno M, Piscitelli P, Colazzo S, Colao A, Miani A. School as ideal setting to promote health and wellbeing among young people. *Health Promot Perspect*. 2020;10(4):316-324. Published November 7, 2020. doi:10.34172/hpp.2020.50
28. Vought-O’Sullivan V, Meehan NK, Havice PA, Pruitt RH. Continuing Education: A National Imperative for School Nursing Practice. *J of Sch Nurs*. 2006;22(1):2-8. doi:10.1177/10598405060220010201
29. Hoke AM, Keller CM, Grimm CL, Lehman EB, Sekhar DL. Impact of Wellness Policy Review, Wellness Council Activity, and Student Health Objectives on Overall School Wellness Climate. *J Sch Health*. 2024;94(3):235-242. doi:10.1111/JOSH.13324
30. CDC Healthy Schools – Parents for Healthy Schools. Centers for Disease Control and Prevention. Published online July 19, 2024. Accessed October 28, 2025. https://www.cdc.gov/healthy-schools/parents/index.html
31. CDC – Local School Wellness Policy. Centers for Disease Control and Prevention. Published online July 24, 2024. Accessed October 28, 2025. https://www.cdc.gov/assessing-improving-school-health/wellness/index.html#:~:text=Local%20school%20wellness%20policy%20requirements,the%20local%20school%20wellness%20policy.
